# Nonlinear electrical impedance spectroscopy of viruses using very high electric fields created by nanogap electrodes

**DOI:** 10.3389/fmicb.2015.00940

**Published:** 2015-09-09

**Authors:** Ryuji Hatsuki, Ayae Honda, Masayuki Kajitani, Takatoki Yamamoto

**Affiliations:** ^1^Department of Mechanical and Control Engineering, Tokyo Institute of TechnologyTokyo, Japan; ^2^Faculty of Bioscience and Applied Chemistry, Housei UniversityTokyo, Japan; ^3^Department of Bioscience, Teikyo UniversityTochigi, Japan

**Keywords:** virus, virus sensing, impedance spectroscopy, nanogap, nanofluidics, environmental monitoring

## Abstract

Our living sphere is constantly exposed to a wide range of pathogenic viruses, which can be either known, or of novel origin. Currently, there is no methodology for continuously monitoring the environment for viruses in general, much less a methodology that allows the rapid and sensitive identification of a wide variety of viruses responsible for communicable diseases. Traditional approaches, based on PCR and immunodetection systems, only detect known or specifically targeted viruses. We here describe a simple device that can potentially detect any virus between nanogap electrodes using nonlinear impedance spectroscopy. Three test viruses, differing in shape and size, were used to demonstrate the general applicability of this approach: baculovirus, tobacco mosaic virus (TMV), and influenza virus. We show that each of the virus types responded differently in the nanogap to changes in the electric field strength, and the impedance of the virus solutions differed depending both on virus type and virus concentration. These preliminary results show that the three virus types can be distinguished and their approximate concentrations determined. Although further studies are required, the proposed nonlinear impedance spectroscopy method may achieve a sensitivity comparable to that of more traditional, but less versatile, virus detection systems.

## Introduction

Our environment is posed a constant threat of exposure by pathogenic viruses, whether to humans or pets, domestic animals, or agricultural and marine products (plants, fish, or shellfish). No established methodology exists for inactivating most viruses that could cause widespread communicable viral infection. It is therefore imperative to realize a technology that can monitor the environment for viruses and prevent their spread and disease transmission. The key is to develop a practical sensor that can continuously monitor the environment for viruses. Such virus sensors could be installed at airports, seaports, and other points of entry, and where necessary at farms and ranches, at restaurants, in air conditioning units, in sewage systems, and in other public facilities or utilities. These sensors would continuously monitor for viruses, and if they were detected, measures could be implemented to prevent infection, in contrast to their existing diagnostic application. In cases where detection and countermeasures are too late to prevent an outbreak of a communicable disease, a network of virus sensors could facilitate rapid identification of affected areas, allowing medical resources to be focused in those areas. This would lead to early-stage detection and treatment, and maximize the efficient use of medical resources.

Currently, there is no technology that can sense viruses in our daily environment. Existing virus detection methods are focused on diagnosing infected individuals, and can be classified into three main categories: (1) those that utilize genomic information and are based on the polymerase chain reaction (PCR) and metagenomic analysis; (2) those that utilize molecular recognition or receptor linking functions of compounds such as antibodies, sugar chains, and peptide aptamers; and (3) those that are based on the electrical or optical properties of viruses (Cheng et al., 2009). In the first category, PCR-based methods provide high detection sensitivity and accuracy but require long analysis times, costly equipment, and specialized techniques, so their range of use is limited in terms of location and qualified personnel (Yang and Rothman, 2004; Espy et al., 2006; Charlton et al., 2009; Hodneland et al., 2011; Cella et al., 2013). In the second category, immunochromatography can provide a relatively fast, simple, and portable means of virus detection in clinical settings such as hospitals, and has therefore become a mainstream technique; however, it suffers from problems such as low sensitivity and accuracy that impede early-stage detection (Lee et al., 1993, 2009; Patolsky et al., 2004; Reichmuth et al., 2008; Heinze et al., 2009; Wang et al., 2009, 2011; Hassen et al., 2011; Lum et al., 2012; Nguyen et al., 2012). Both categories, moreover, basically involve single-use disposable test units, and can only detect known or specifically targeted viruses. In short, neither category is appropriate in principle for application to continuous virus monitoring of the environment, or detecting new or unspecified viruses. Consequently, neither category holds significant promise for the development of a systematic technology for comprehensive environmental virus monitoring.

There have been fewer studies in the third category, which are based on direct measurement of physical properties. The methods in this category are inferior to the other two in terms of sensitivity, and are particularly poor in their ability to selectively distinguish viruses in the presence of numerous contaminants. However, methods in the third category have two key potential advantages: the ability to continuously monitor the environment, and to detect unspecified viruses and mutants, since biological information on genomes or receptors is not required. A high-sensitivity, accurate virus sensor based on direct measurement of physical properties would enable long-term, continuous virus monitoring, which cannot be achieved by the other two categories. This would represent a groundbreaking advance in virus-monitoring technology.

Most virus detection techniques based on physical properties use optical or electrical methods. Typical optical methods use light scattering to achieve high-sensitivity detection to confirm the presence of virus particles. However, optical detection has disadvantages in miniaturization of a complex optical setup and light source (Fan et al., 2008). On hand, electrical method for direct virus detection uses impedance spectroscopy, while another uses dielectric relaxation (Ermolina et al., 2003). Other recent examples include microelectrodes fabricated in microchannels coupled with impedance spectroscopy. This approach is aimed at identifying and quantifying baculovirus and lentivirus in solutions, based on the impedance magnitude at the peak frequency (Poenar et al., 2004). However, the accuracy and sensitivity of these approaches are low compared with PCR-based and immunochromatographic methods, and there have been few publications regarding these techniques.

We investigated the use of impedance spectroscopy for highly sensitive and accurate virus detection based on the nonlinear effect of electrophoretic and dielectrophoretic forces on the virion during measurement to enhance both sensitivity and selectivity. The approach involves applying a sufficiently strong electric field to cause inter-electrode virion movement and allow control of the orientation in the case of non-spherical virions. This active perturbation of the virion allows nonlinear measurement of its properties.

Generally, an electric field of 10^5^ V/m or more is required to induce movement of nanometer-sized particles by dielectrophoretic forces, but an applied voltage of several volts or more ordinarily required for this purpose would also induce prominent electrode reactions that would tend to preclude effective for measurement (Turcu and Lucaciu, 1989; Akin et al., 2004; Liu and Bau, 2004; Yang et al., 2008; Pethig, 2010). To obtain an electric field of 10^5^ V/m or more using a lower applied voltage, we propose the use of nanogap electrodes. For example, it is possible to generate an electric field of 200 kV/m across a 500 nm gap using an applied voltage of 0.1 V, which is too low for electrolysis to occur. This study reports a high-sensitivity virus detection method by nonlinear impedance spectroscopy with the use of nanogap electrodes to generate strong electric fields under a low applied voltage.

## Materials and methods

### Virus samples

Table 1 shows the shapes and sizes of the three viruses used in this study. The dipole moment induced in a small particle generally depends on its size and shape, and the dipolar effect tends to be particularly strong for long, narrow particles. We therefore used baculovirus, tobacco mosaic virus (TMV), and influenza virus [influenza A (H1N1)] as sample viruses with different sizes and long/short axis ratios. The baculovirus is rod-shaped, with a diameter of 30–60 nm and a length of approximately 260 nm (Burley et al., 1982; Choi et al., 2012). The TMV is approximately 20 nm in diameter and 300 nm in length (Klug, 1999), and the influenza A (H1N1) virus is spherical with a diameter of approximately 100 nm (Tiffany and Blough, 1970; Sai et al., 2011).

**Table 1:** **Dimensions of viruses used in this study**.

**Virus**	**Shape**	**Size**
Influenza	Sphere	100 nm
TMV	Rod	20 nm diameter and 300 nm length
Baculo	Rod	30–60 nm diameter and 260 nm length

### Quantification of virus

**Figure 1:**
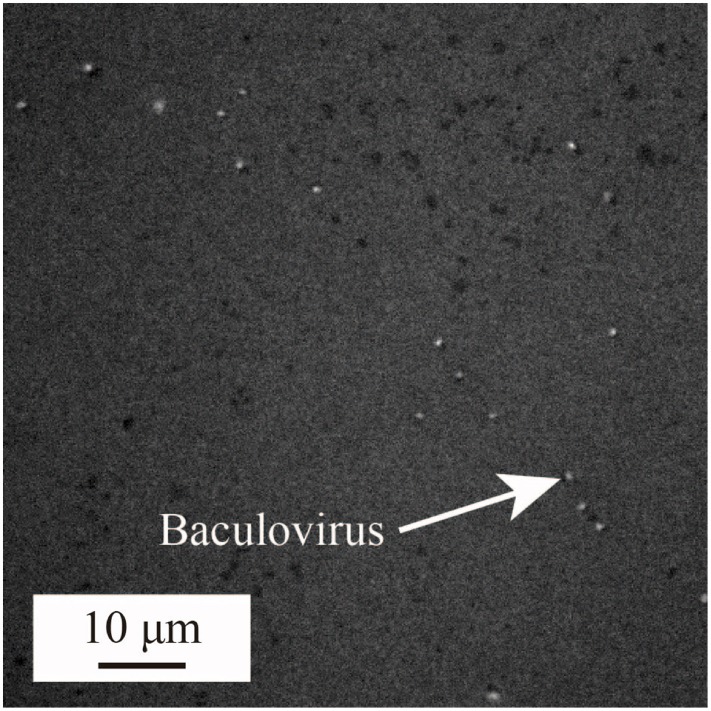
**Scanning electron microscopy of baculovirus used for counting the number of virions**.

To date, virus concentrations is generally determined based on the tissue culture infective dose (TCID50) or plaque forming units (PFU) leaving the number of virions is unclear, making it difficult to quantify the sensitivity of the sensing and to compare the results with those of other methods. To prevent this problem, we first visualized and counted the number of virions by fluorescence labeling and observation, then expressed the concentration in terms of virions per unit volume. 1 mM KCl buffer was used, and a fluorescent dye (SYBR-GOLD, Invitrogen Inc.) was added to solution for labeling the viruses. The labeled virions were counted under a fluorescence microscope. Figure 1 shows a typical result, with each bright spot representing a single baculovirion. The virion concentrations were estimated from the bright spot counts using image analysis software (Image J) (Schneider et al., 2012).

### Device fabrication

**Figure 2:**
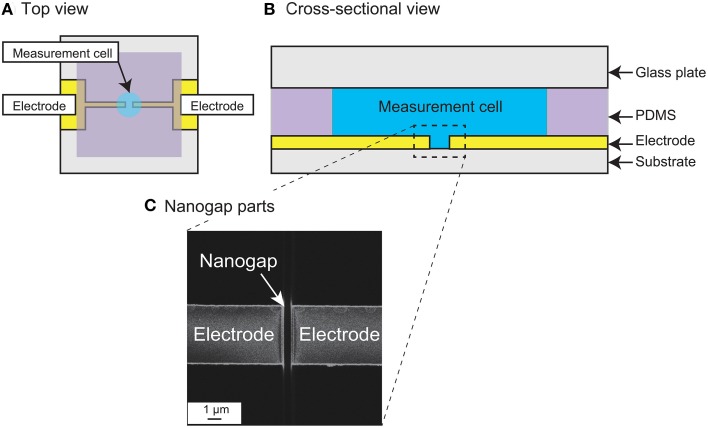
**(A)** Schematic top view and **(B)** Cross-sectional view of the measurement device with nanogap electrodes. **(C)** Scanning ion microscopy image of the measurement region, showing the nanogap electrodes. The gap width is 510 nm.

Figures 2A,B show top and cross-sectional schematic views of the measurement device. The device is basically fabricated on a quartz substrate patterned with two nanogap electrodes, a polydimethylsiloxane (PDMS) sheet forming the measurement chamber wall, and a glass plate as the chamber lid. The nanogap electrodes were patterned with an Au (250 nm)/Ti (1 nm) layer in strip fabricated by photolithographic lift-off, and the strip was then cut to form opposing electrodes with an intervening nanogap using a focused ion beam (FIB; FB-2200, Hitachi High-Technologies Corp.) (Hatsuki et al., 2013). Figure 2C shows a scanning ion micrograph of the fabricated measurement region. The small grooves in the quartz substrate between and on both sides of the nanogap are overrun regions for cutting the parallel flat-plate electrodes in the FIB process. The fabricated gold/metal electrodes were 250 nm in height and 5 μm in width, with an intervening gap width of 510 nm. The depth of the groove was about 530 nm in the middle of the nanogap. A hole with a diameter of 3 mm was opened in the 0.2-mm-thick PDMS sheet to form the wall of the cell chamber (approximately 1.5 μL volume) and the sheet was then bonded in position to form the measurement cell. For the impedance measurements, the chamber was filled with the sample solution and closed with the glass lid.

### Impedance measurements

The impedance was measured using a frequency response analyzer (1260, Solartron Analytical) with a dielectric interface (1296, Solartron Analytical), which were controlled with SMART software (Solartron Analytical), and the data were analyzed using Zview software (Solartron Analytical).

The electrical impedance of liquid samples suffer from several problems, including unavoidable electrode decomposition and substantial changes in the conductivity due to the release of ions, and damages to the virus with these ions in high concentrations. These problems were apparently appeared in low frequency region less than 100 kHz in our preliminary measurements, so that the measurements were conducted in the range 100 kHz–6.3 MHz. Prior to each measurement, open/short calibration was performed, and the effects of parasitic capacitance and other parasitic components, wiring induction coupling, and leakage were eliminated. The applied frequency for all of the measurements was 100 kHz or higher, to prevent background effects from the solid-liquid double layer capacitance that tends to form at the interface of the measuring electrode (Poenar et al., 2004); this layer is large at low frequencies but minimal at high frequencies. The virion impedance component was obtained by subtracting the separately measured impedance component for the 1 mM KCl buffer solution from the total solution impedance; corrected measurements were used in all assessments. Because the repeated measurement of the same sample shows no apparent difference in electrical signal, it is assumed that the application of high electric field of 10^5^ V/m or more would not be significantly damaging to the virus. It is, however, not confirmed whether the effect of high electric field is how much hazardous to the viruses in their biological activities.

## Results and discussion

### Impedance dependence on electric field strength

We first investigated the presence of a nonlinear impedance effect in a strong electric field by measuring the impedance while increasing the electric field strength incrementally from 10^4^ V/m to 2 × 10^5^ V/m, using a baculovirus solution with 10^14^ virions/mL.

**Figure 3:**
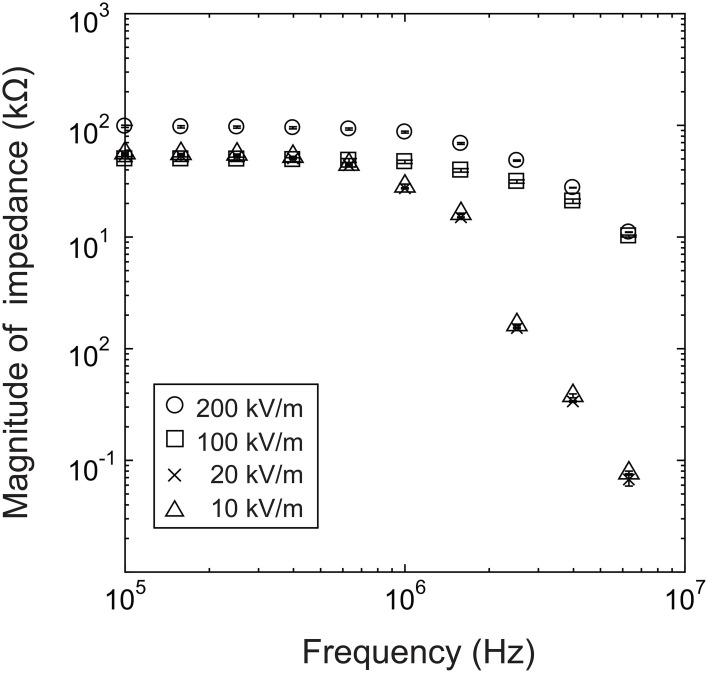
**Dependence of impedance response on electric field strength**.

As shown in Figure 3, at frequencies above 1 MHz, the impedance response is very different for an electric field of 100 kV/m (50 mV) or greater than for an electric field of 20 kV/m (10 mV) or less. This is presumably at least partially attributable to the desired effect of the strong nonlinear electric field and the related dielectrophoretic force that generally arises in strong high-frequency electric fields. Many studies have shown that positive dielectrophoresis (in which the force is exerted toward higher field strength) occurs in electric fields of several 100 kV/m to several MV/m with frequencies of several 100 kHz or higher (Morgan and Green, 1997; Morgan et al., 1999; Hughes et al., 2001; Park et al., 2007). This suggests that the virions between the electrodes were moved by this force and aligned their long axes with the field direction due to the torque by the electric field. In some cases, the virions adhered to the electrode edges, where the field strength was maximum in the measurement area, resulting in a nonlinear increase in impedance. However, it was not possible to confirm this mechanism because the excitation light required for fluorescence measurements during the impedance measurements would have introduced noise into the system. At present, therefore, this explanation remains speculation.

In summary, the measurements showed changes in impedance that are presumably a nonlinear effect of an applied electric field of 100 kV/m or higher at a frequency above 1 MHz. In light of these results, we used a field strength of 200 kV/m (100 mV) in the subsequent experiments.

The electric field was designed by the finite element method using the commercially available solver COMSOL 4.4 (COMSOL Inc.). Based on simulation, the region between the electrodes for an electric field of 100 kV/m or higher was obtained with a gap length of 510 nm, an electrode width of 5 μm, and a depth of groove approximately 530 nm, corresponding to a volume of about 1.4 fL. This is a rough estimate due to the errors inherent in the measurements; regardless, for a sample concentration of 10^11^ virions/mL, this volume would contain approximately 0.3 virions. Under the assumption that the number of virions measured with a sample concentration of 10^11^ virions/mL would actually be one to several, we set 10^11^ virions/mL concentration as the lower limit for subsequent experiments.

### Impedance dependence on virus concentration

**Figure 4:**
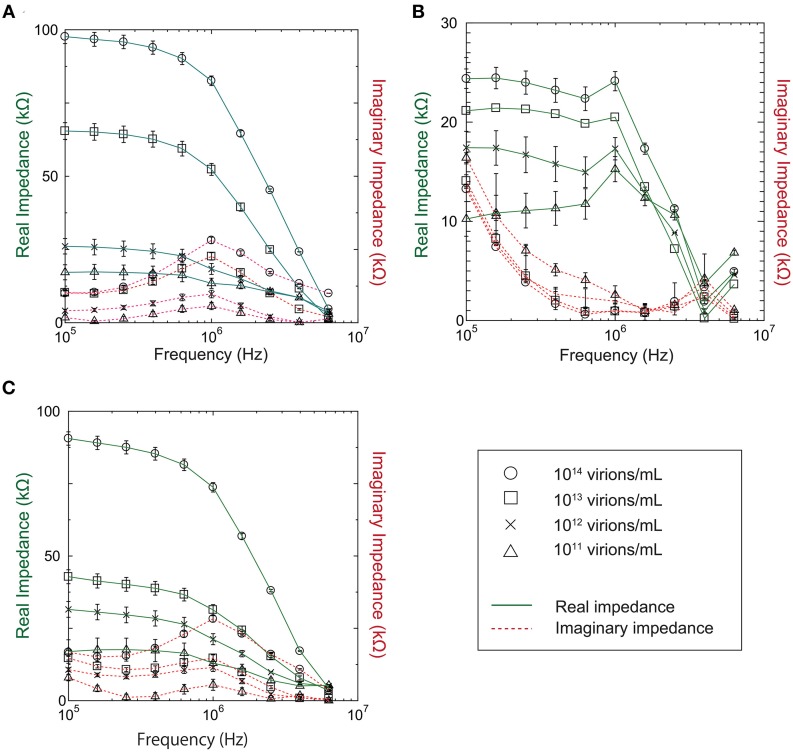
**Real and imaginary components of measured impedance for (A) Baculovirus, (B) TMV, and (C) Influenza virus**.

Our investigation of the impedance dependence on virus concentration showed that the impedance varies with virus concentration in the range 10^11^–10^14^ virions/mL for baculovirus, TMV, and influenza virus, as shown in Figure 4. In the figure, the solid and dotted lines represent the real and imaginary impedance components, respectively. For the real component, similar behavior is found for the baculovirus and influenza viruses, as shown in Figure 4A and Figure 4C, respectively. The values decrease moderately with increasing frequency between 100 kHz and 1 MHz, then fall sharply at higher frequency, and also generally increase with increasing sample concentration. In contrast, the imaginary component for both viruses shows a peak near 1 MHz that increases in height with increasing sample concentration. As shown in Figure 4C, however, the trends exhibited by TMV clearly differ from those for the baculovirus and influenza viruses. The real impedance component for TMV peaks at 1 MHz, then decreases rapidly at higher frequency. The values generally increase with increasing sample concentration as with the other two viruses, whereas the imaginary impedance component for TMV tends to decrease up to a frequency of 1 MHz, and then increase to a maximum near 3.9 MHz.

**Figure 5:**
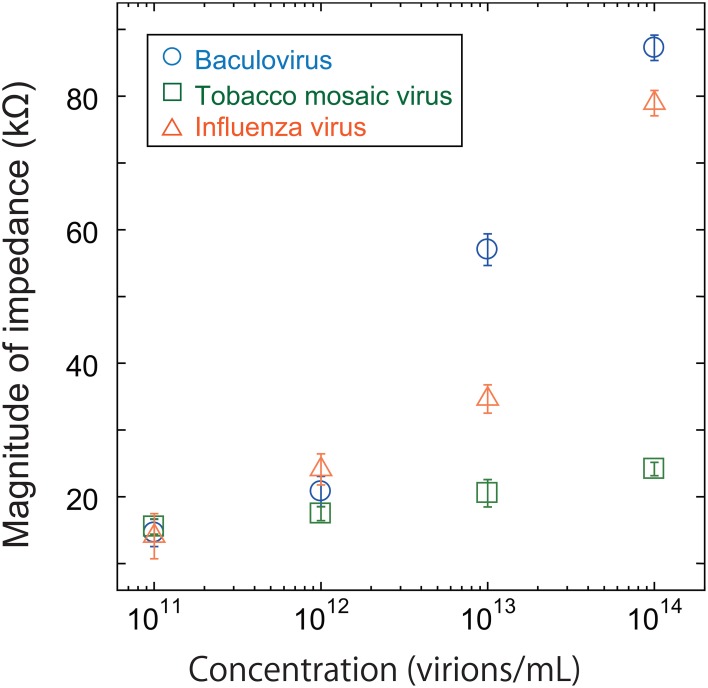
**Magnitude of impedance for baculovirus, TMV, and influenza virus solution at 1 MHz**. The concentration was varied from 10^11^ to 10^14^ virions/mL.

As shown in Figure 4, all three viruses showed an impedance peak and transition point near 1 MHz for both the real component and imaginary components. In Figure 5, the vertical and horizontal axes represent the magnitude of the impedance and the virus concentration, respectively. The figure shows the dependence of the impedance magnitude at 1 MHz on the virus concentration for each virus. These results clearly indicate that the impedance values for the three virus types tend to increase with increasing concentration and can therefore be used to quantify the virus concentration.

### Distinguish virus types

It would be difficult to distinguish quantitatively between the three virus types using only the impedance spectra shown in Figure 4. The baculovirus and influenza virus spectra are similar, although they are both distinct from that of TMV.

**Figure 6:**
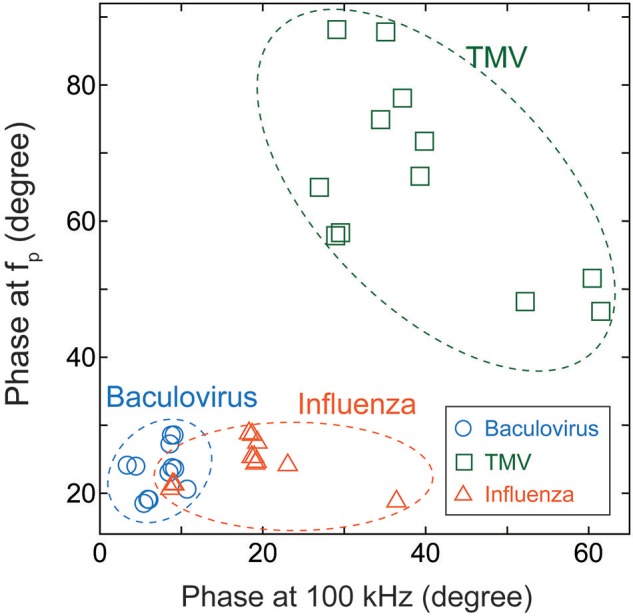
**Cluster map of Baculovirus, TMV, and influenza virus**. The data were obtained for virus concentrations of 10^11^–10^14^ virions/mL, and plotted with the phase at 100 kHz along the horizontal axis and the phase at the peak frequency of the imaginary component of impedance along the vertical axis.

We therefore compared the three virus types, and particularly the baculovirus and influenza virus, for differences in phase effects. Figure 6 shows the mapped the data obtained for each virus at concentrations of 10^11^–10^14^ virions/mL. The horizontal and vertical axes represent the phases of the imaginary impedance component somewhat arbitrarily at 100 kHz and the peak frequency, f_p_, respectively. Although some overlap is apparent between the three clusters of data points, the graph clearly shows that it is possible to distinguish between virus types on this basis, independent of their concentrations.

It should be noted, however, that this investigation was performed for only these three viruses, and it would be premature to extrapolate the results to other virus types.

## Conclusions

Here we proposed a virus detection method by nonlinear impedance spectroscopy under a strong electric field between two nanogap electrodes.

In sweeping frequencies from 100 Hz to 6.3 MHz while varying the electric field strength, we found an apparently marked nonlinear effect on impedance at electric field strengths of 100 kV/m or higher. Further measurements were performed for those electric field strengths and frequency while varying the concentrations of baculovirus, TMV, and influenza A (H1N1) virus solutions. The data clearly showed that the virus concentration can be quantified by the impedance value. Furthermore, the three virus types could be distinguished by plotting the phase at 100 kHz against the phase of the peak value of the imaginary component of the impedance for each virus type.

At the minimum sample concentration of 10^11^ virions/mL used in this study, one to several virions occupied the effective measurement space. This is a rough estimate, and a more detailed study is required for verification. The results nevertheless show that the proposed nonlinear impedance spectroscopy method may achieve a sensitivity comparable to that of PCR and immunodetection systems. The response time to sweep the frequency for each measurement was about a few minutes, which will be enough fast for the continuous monitoring of environment. One of the large remaining issues is to evaluate the detection and identification ability of some specific viruses from the heterogeneous mixture of crude sample. That is the universal challenge for any types of biosensing methods, and also the future work to put this method into practical use.

### Conflict of interest statement

The authors declare that the research was conducted in the absence of any commercial or financial relationships that could be construed as a potential conflict of interest.

## References

[B1] Akin D. Li H. Bashir R. (2004). Real-time virus trapping and fluorescent imaging in microfluidic devices. Nano Lett. 4, 257–259. 10.1021/nl034987p

[B2] Burley S. K. Miller A. Harrap K. A. Kelly D. C. (1982). Structure of the baculovirus nucleocapsid. Virology 120, 433–440. 10.1016/0042-6822(82)90043-5 18638731

[B3] Cella L. N. Blackstock D. Yates M. A. Mulchandani A. Chen W. (2013). Detection of RNA viruses: current technologies and future perspectives. Crit. Rev. Eukaryot. Gene Expr. 23, 125–137. 10.1615/CritRevEukaryotGeneExpr.2013006974 23582035

[B4] Charlton B. Crossley B. Hietala S. (2009). Conventional and future diagnostics for avian influenza. Comp. Immunol. Microbiol. Infect. Dis. 32, 341–350. 10.1016/j.cimid.2008.01.009 18448167

[B5] Cheng X. Chen G. Rodriguez W. R. (2009). Micro- and nanotechnology for viral detection. Anal. Bioanal. Chem. 393, 487–501. 10.1007/s00216-008-2514-x 19052733 PMC7080050

[B6] Choi J. Y. Roh J. Y. Wang Y. Zhen Z. Tao X. Y. Lee J. H.. (2012). Analysis of genes expression of *Spodoptera exigua* larvae upon AcMNPV infection. PLoS ONE 7:e42462. 10.1371/journal.pone.0042462 22860129 PMC3409162

[B7] Ermolina I. Morgan H. Green N. G. Milner J. J. Feldman Y. (2003). Dielectric spectroscopy of tobacco mosaic virus. Biochim. Biophys. Acta 1622, 57–63. 10.1016/S0304-4165(03)00118-1 12829262

[B8] Espy M. J. Uhl J. R. Sloan L. M. Buckwalter S. P. Jones M. F. Vetter E. A.. (2006). Real-time PCR in clinical microbiology: applications for routine laboratory testing. Clin. Microbiol. Rev. 19, 165–256. 10.1128/CMR.19.1.165-256.2006 16418529 PMC1360278

[B9] Fan X. White I. M. Shopova S. I. Zhu H. Suter J. D. Sun Y. (2008). Sensitive optical biosensors for unlabeled targets: a review. Anal. Chim. Acta. 6, 8–26. 10.1016/j.aca.2008.05.022 18558119 PMC10069299

[B10] Hassen W. M. Duplan V. Frost E. Dubowski J. J. (2011). Quantitation of influenza A virus in the presence of extraneous protein using electrochemical impedance spectroscopy. Electrochim. Acta 56, 8325–8328. 10.1016/j.electacta.2011.07.009

[B11] Hatsuki R. Fuchigami Y. Yamamoto T. (2013). Direct measurement of electric double layer in a nanochannel by electrical impedance spectroscopy. Microfluid. Nanofluid. 14, 983–988. 10.1007/s10404-012-1105-5

[B12] Heinze B. C. Song J. Y. Lee C. H. Najam A. Yoon J. Y. (2009). Microfluidic immunosensor for rapid and sensitive detection of bovine viral diarrhea virus. Sens. Actuators B Chem. 138, 491–496. 10.1016/j.snb.2009.02.058

[B13] Hodneland K. García R. Balbuena J. A. Zarza C. Fouz B. (2011). Real-time RT-PCR detection of betanodavirus in naturally and experimentally infected fish from Spain. J. Fish Dis. 34, 189–202. 10.1111/j.1365-2761.2010.01227.x 21306586

[B14] Hughes M. P. Morgan H. Rixon F. J. (2001). Dielectrophoretic manipulation and characterization of herpes simplex virus-1 capsids. Eur. Biophys. J. 30, 268–272. 10.1007/s002490100144 11548129

[B15] Klug A. (1999). The tobacco mosaic virus particle: structure and assembly. Philos. Trans. R. Soc. Lond. B Biol. Sci. 354, 531–535. 10.1098/rstb.1999.0404 10212932 PMC1692534

[B16] Lee B. W. Bey R. F. Baarsch M. J. Simonson R. R. (1993). ELISA method for detection of influenza A infection in swine. J. Vet. Diagn. Invest. 5, 510–515. 10.1177/104063879300500402 8286447

[B17] Lee Y. F. Lien K. Y. Lei H. Y. Lee G. B. (2009). An integrated microfluidic system for rapid diagnosis of dengue virus infection. Biosens. Bioelectron. 25, 745–752. 10.1016/j.bios.2009.08.020 19744849 PMC7125828

[B18] Liu H. Bau H. H. (2004). The dialectrophoresis of cylindrical and spherical particles submerged in shells and in semi-infinite media. Phys. Fluids 16, 1217–1228. 10.1063/1.1649237

[B19] Lum J. Wang R. Lassiter K. Srinivasan B. Abi-Ghanem D. Berghman L.. (2012). Rapid detection of avian influenza H5N1 virus using impedance measurement of immuno-reaction coupled with RBC amplification. Biosens. Bioelectron. 38, 67–73. 10.1016/j.bios.2012.04.047 22647532

[B20] Morgan H. Green N. G. (1997). Dielectrophoretic manipulation of rod-shaped viral particles. J. Electrostat. 40, 279–293. 10.1016/S0304-3886(97)00159-99350515

[B21] Morgan H. Hughes M. P. Green N. G. (1999). Separation of submicron bioparticles by dielectrophoresis. Biophys. J. 77, 516–525. 10.1016/S0006-3495(99)76908-0 10388776 PMC1300348

[B22] Nguyen B. T. Peh A. E. Chee C. Y. Fink K. Chow V. T. Ng M. M.. (2012). Electrochemical impedance spectroscopy characterization of nanoporous alumina dengue virus biosensor. Bioelectrochemistry 88, 15–21. 10.1016/j.bioelechem.2012.04.006 22763420

[B23] Park K. Akin D. Bashir R. (2007). Electrical capture and lysis of vaccinia virus particles using silicon nano-scale probe array. Biomed. Microdevices 9, 877–883. 10.1007/s10544-007-9101-3 17610069

[B24] Patolsky F. Zheng G. Hayden O. Lakadamyali M. Zhuang X. Lieber C. M. (2004). Electrical detection of single viruses. Proc. Natl. Acad. Sci. U.S.A. 101, 14017–14022. 10.1073/pnas.0406159101 15365183 PMC521090

[B25] Pethig R. (2010). Dielectrophoresis: status of the theory, technology, and applications. Biomicrofluidics 4, 022811 10.1063/1.3456626 20697589 PMC2917862

[B26] Poenar D. P. Iliescu C. Boulaire J. Yu H. (2004). Label-free virus identification and characterization using electrochemical impedance spectroscopy. Electrophoresis 35, 433–440. 10.1002/elps.201300368 24285469

[B27] Reichmuth D. S. Wang S. K. Barrett L. M. Throckmorton D. J. Einfeld W. Singh A. K. (2008). Rapid microchip-based electrophoretic immunoassays for the detection of swine influenza virus. Lab Chip 8, 1319–1324. 10.1039/b801396a 18651074

[B28] Sai L. Frederic E. Christian S. Andreas H. Iwan A. T. Schaap I. A. (2011). Bending and puncturing the influenza lipid envelope. Biophys. J. 100, 637–645. 10.1016/j.bpj.2010.12.3701 21281578 PMC3030173

[B29] Schneider C. A. Rasband W. S. Eliceiri K. W. (2012). NIH image to image J: 25 years of image analysis. Nat. Methods 9, 671–675. 10.1038/nmeth.2089 22930834 PMC5554542

[B30] Tiffany J. M. Blough H. (1970). Models of structure of the envelope of influenza virus. Proc. Natl. Acad. Sci. U.S.A. 65, 1105–1012. 10.1073/pnas.65.4.1105 5266149 PMC283029

[B31] Turcu I. Lucaciu C. M. (1989). Dielectrophoresis: a spherical shell model. J. Phys. A Math. Gen. 22, 985–993. 10.1088/0305-4470/22/8/014

[B32] Wang R. Lin J. Lassiter K. Srinivasan B. Lin L. Lu H.. (2011). Evaluation study of a portable impedance biosensor for detection of avian influenza virus. J. Virol. Methods 178, 52–58. 10.1016/j.jviromet.2011.08.011 21872621

[B33] Wang R. Wang Y. Lassiter K. Li Y. Hargis B. Tung S.. (2009). Interdigitated array microelectrode based impedance immunosensor for detection of avian influenza virus H5N1. Talanta 79, 159–164. 10.1016/j.talanta.2009.03.017 19559858

[B34] Yang L. Banada P. P. Bhunia A. K. Bashir R. (2008). Effects of dielectrophoresis on growth, viability and immuno-reactivity of listeria monocytogenes. J. Biol. Eng. 2:6. 10.1186/1754-1611-2-6 18416836 PMC2373775

[B35] Yang S. Rothman R. E. (2004). PCR-based diagnostics for infectious diseases: uses, limitations, and future applications in acute-care settings. Lancet Infect. Dis. 4, 337–348. 10.1016/S1473-3099(04)01044-8 15172342 PMC7106425

